# Activation of protein kinase A in the amygdala modulates anxiety-like behaviors in social defeat exposed mice

**DOI:** 10.1186/s13041-015-0181-3

**Published:** 2016-01-08

**Authors:** Liu Yang, Li-Jun Shi, Jin Yu, Yu-Qiu Zhang

**Affiliations:** Institute of Neurobiology, Institutes of Brain Science and State Key Laboratory of Medical Neurobiology, Collaborative Innovation Center for Brain Science, Fudan University, 1202 Mingdao Building, 131 Dong An Road, Shanghai, 200032 China; Department of Integrative Medicine and Neurobiology, Shanghai Medical School, Fudan University, Shanghai, 200032 China

**Keywords:** Social defeat, Anxiety, PKA, CREB, Amygdala

## Abstract

**Background:**

Social defeat (SD) stress induces social avoidance and anxiety-like phenotypes. Amygdala is recognized as an emotion-related brain region such as fear, aversion and anxiety. It is conceivable to hypothesize that activation of amygdala is involved in SD-dependent behavioral defects.

**Results:**

SD model was established using C57BL/6J mice that were physically defeated by different CD-1 mice for 10 days. Stressed mice exhibited decreased social interaction level in social interaction test and significant anxiety-like behaviors in elevated plus maze and open field tests. Meanwhile, a higher phosphorylation of PKA and CREB with a mutually linear correlation, and increased Fos labeled cells in the basolateral amygdala (BLA) were observed. Activation of PKA in the BLA by 8-Br-cAMP, a PKA activitor, significantly upregulated pCREB and Fos expression. To address the role of PKA activation on SD stress-induced social avoidance and anxiety-like behaviors, 8-Br-cAMP or H-89, a PKA inhibitor, was continuously administered into the bilateral BLA by a micro-osmotic pump system during the 10-day SD period. Neither H-89 nor 8-Br-cAMP affected the social behavior. Differently, 8-Br-cAMP significantly relieved anxiety-like behaviors in both general and moderate SD protocols. H-89 per se did not have anxiogenic effect in naïve mice, but aggravated moderate SD stress-induced anxiety-like behaviors. The antidepressant clomipramine reduced SD-induced anxiety and up-regulated pPKA level in the BLA.

**Conclusions:**

These results suggest that SD-driven PKA activation in the basolateral amygdala is actually a compensatory rather than pathogenic response in the homeostasis, and modulating amygdaloid PKA may exhibit potency in the therapy of social derived disorders.

**Electronic supplementary material:**

The online version of this article (doi:10.1186/s13041-015-0181-3) contains supplementary material, which is available to authorized users.

## Background

Anxiety disorders are the most common of all mental health problems that affect human beings. Over the past several decades, numerous investigations with human subjects have demonstrated a close correclation between stressful life events and anxiety [1]. Moreover, a large body of animal studies has revealed striking parallels in the neurobiological abnormalities caused by stress and those found in anxiety/depressive patients [2]. Social derived stresses account for the highest proportion in today's psychotogenic stresses [3, 4]. Social defeat (SD) stress obtained in the resident/intruder paradigm has been reported to cause a variety of molecular, physiological, and behavioral changes [5, 6]. In particular, SD stress in animals induces social avoidance, anxiety- and depression-like phenotype [7–9]. Hence, SD paradigm with accepted ethological validity has been applied to imitate the social stress-related disorders of human society.

Accumulating evidence suggests that the amygdala is involved in fear and anxiety [10–14]. In patients with PTSD, increased amygdaloid activation is associated with symptoms such as fear, social avoidance, anxiety and depression [15]. Social stress-induced fear and anxiety-like behaviors are correclated with Fos expression [16, 17] and dendritic hypertrophy [18] in the amygdala. Inhibition of glutamatergic signaling in the basolateral amygdala (BLA) blocks anxiety responses as measured by social preference [19]. Furthermore, upregulating amygdaloid cAMP responsive elementbinding protein (CREB), a transcription factor, alleviate anxiety-like behaviors [20, 21]. It is well known that CREB is regulated via phosphorylation at serine 133 by cAMP-dependent protein kinase A (PKA) [22, 23], and cAMP-PKA signaling in molecular pathways is involved in anxiety and formation of fear memory [24]. Despite the role of amygdala and PKA signaling in fear and anxiety-like behaviors, little is known about PKA activation in this area and its impact on anxiety by social stress. We hypothesized that activation of amygdaloid PKA may be involved in SD-dependent behavioral defects. In this study, using a mouse SD stress model that allows simultaneous measurement of social avoidance and anxiety-like behaviors, we demonstrated that activation of PKA in the BLA plays an anti-anxiety but not counter-social avoidance role.

## Results

### Social defeat stress induces social avoidance and anxiety-like behaviors

**Fig. 1:**
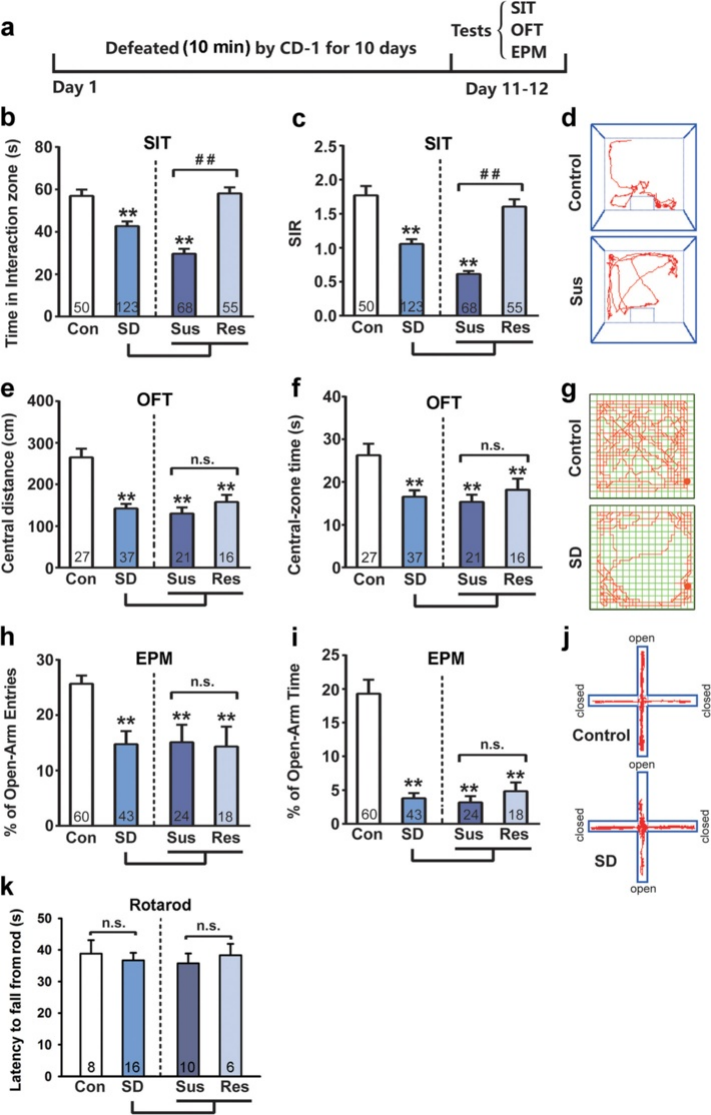
Social defeat (SD) stress induced social avoidance and anxiety-like behaviors. **a** Schematic representation of the protocols of SD and behavioral tests; **b**-**d** SD stress induced social avoidance in the social interaction test (SIT). Either susceptible individuals or the total pool (susceptible + resilient), the time in the social interaction zone (**b**) and the social interaction ratio (**c**) was lower than controls; **d** is the representative trajectories of a control and a susceptible mice in the social interaction test. **e**-**g** In the open field test (OFT), SD stress decreases central-zone distance (**e**) and time (**f**) in both susceptible and resilient mice; **g** is the representative trajectories in the OFT; **h**-**j** In the elevated plus maze (EPM) test, SD stress decreased percentages of open-arm entries (**h**) and time (**i**) in both susceptible and resilient mice. **j** is the representative trajectories in elevated plus maze. **k** SD stress did not influence motor coordination in a rotarod test. *p < 0.05, **p < 0.01 verse control.

C57BL/6J mice that underwent a 10-day SD protocol displayed a reduction in social interaction, which is measured by comparing the time a mouse spent in an interaction zone (IZ) with a social mouse (CD1) to the time in that zone in the absence of a social mouse. Defeated mice were divided into susceptible and resilient groups according to whether they exhibited social avoidance (susceptible: social interaction ratio, SIR < 1) or social preference (resilient: SIR ≥ 1). As shown in Fig. 1a-d, 68 of 123 mice spent significant less time in IZ in the presence of a social mouse with a SIR less than 1 (Susceptible). Almost all the control mice not previously exposed to an aggressive CD1 had SIRs greater than 1 (Fig. 1c). When all susceptible and resilient mice were pooled together, a shorter interaction duration (in target present) and a lower SIR were still revealed as compared to the control group (Fig. 1b and c Student’s *t*-test, *p* < 0.01, SD vs. control. Interaction duration: *t*_*171*_ = 3.535; SIR: *t*_*171*_ = 5.112).

Open field test (OFT) and elevated plus maze (EPM) test were used to assess anxiety-like behaviors. In the OFT, the SD mice, either susceptible or resilient, traveled shorter distances and spent less time in the central zone as compared to control mice (One-way ANOVA, Central-zone distance: F_2, 61_ = 15.53, *p* < 0.01; Central-zone time: F_2, 61_ = 5.909, *p* < 0.01). No significant difference was found between susceptible and resilient mice (Fig. 1e-g). Pooled susceptible and resilient together as SD group, there were significant differences in central distance and central time between SD and control mice (Student’s *t*-test central distance: *t*_*62*_ = 5.496, *p* < 0.01; central time: *t*_*62*_ = 3.368, *p* < 0.01).

Consistent with the OFT, EPM test showed that SD mice had a decreased open-arm preference without difference between susceptible and resilient subgroups (Fig. 1h-j). Both open-arm entries and time in SD exposed mice were significantly lower than that in control mice (One-way ANOVA, percentage of open-arm entries: F_2, 99_ = 8.915, *p* < 0.01; percentage of open-arm time: F_2, 99_ = 18.08, *p* < 0.01). These data suggest that a 10-day SD exposure produces an anxiety-like phenotype that is independent on the social behavior. We therefore pooled averages of susceptible and resilient mice as a SD group in the following experiments.

To rule out the influence of locomotor activity on behavioral tests in SIT, OFT and EMP, a rotarod test was performed to explore the potential motor impairment by 10-day SD exposure. As shown in Fig. 1k, no difference in the mean latency to fall from the rod was found between control and SD mice (Student’s *t*-test, *t*_*22*_ = 0.459, *p* = 0.650).

### Clomipramine relieves social avoidance and anxiety-like behavior

**Fig. 2:**
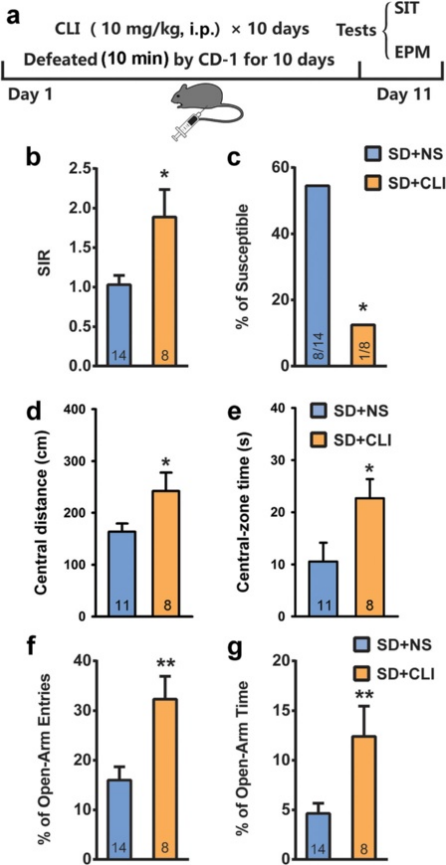
Clomipramine (CLI) relieved social avoidance and anxiety-like behavior. **a** Schematic representation of the SD and CLI administration protocol; **b** & **c** I.p. injection of CLI (10 mg/kg) increased the SIR (**b**) and decreased proportion of susceptible individuals (**c**) compared to the normal saline (NS) in the social interaction test (SIT); **d** & **e** CLI reversed SD stress-induced decreases in central-zone distance (**d**) and time (**e**) in open field test (OFT); **f** & **g** CLI reversed SD stress-induced decreases in both open-arm entries and time in elevated plus maze (EPM) test. *p < 0.05, **p < 0.01 verse NS control.

To confirm SD stress-induced anxiety, a well-characterized antidepressant clomipramine (CLI) was administered 1 h after defeat every day during the 10-day SD period (Fig. 2a). Repeated injections of CLI (10 mg/kg, i.p.) [25, 26] markedly increased the interaction time and SIR of defeated mice (Fig. 2b, Student’s *t*-test, *t*_*20*_ = 2.321, *p* < 0.05). Moreover, the proportion of susceptible individuals to SD stress was significantly decreased by 10-day CLI treatment (Fig. 2c, Fisher's exact test *p* < 0.05). In OFT and EPM test, CLI efficiently reversed SD stress-induced decreases in central distance and central time in OFT (Fig. 2d and e, Student’s *t*-test, central distance: *t*_*17*_ = 2.201, *p* < 0.05; central time: *t*_*17*_ = 2.386, *p* < 0.05), as well as the percentage of open-arm entries and time in EPM test (Fig. 2f and g, Student’s *t*-test, percentage of open-arm entries: *t*_*20*_ = 3.302, *p* < 0.05; percentage of open-arm time: *t*_*20*_ = 2.941, *p* < 0.05).

### Social defeat stress activates PKA and downstream signaling in the BLA

**Fig. 3:**
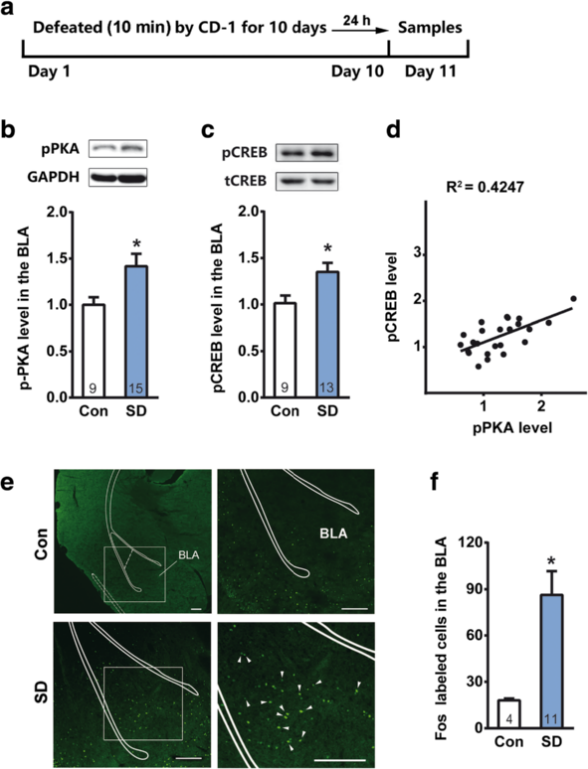
SD stress activated PKA and downstream signaling in the BLA. **a** Schematic representation of the SD and sampling protocol; **b**-**d** SD stress induced PKA (**b**) and CREB (**c**) activation in the BLA; **d** shows a linear correlation between pCREB and pPKA upregulation in the BLA; **e** & **f** Immunofluorescence staining for Fos shows significant increase in number of Fos-positive cells in the BLA. Arrowheads indicate Fos-positive cells, (Scale bar = 100 μm). *p < 0.05 verse control.

Phosphorylation of PKA has been extensively used as an indicator of PKA activation [27, 28]. SD stress induced a robust upregulation of pPKA RII in the basolateral amygdala (BLA) (Fig. 3a and b, Student’s *t*-test, *t*_*22*_ = 2.227, *p* < 0.05). Given that PKA could also facilitate *c-fos* gene expression via phosphorylating the transcription factor CREB [29–31], we therefore examined if SD stress-induced PKA activation correlates with increased pCREB and Fos expression in the BLA. Paralleling with pPKA upregulation, SD stress-induced pCREB were significantly increased in the BLA (Fig. 3a-c, Student’s *t*-test, *t*_*22*_ = 2.483, *p* < 0.05). The increased folds of pPKA with pCREB exhibited a linear correlation (Fig. 3d, Linear regression, pPKA and p-CREB: slope deviation from zero F_1, 22_ = 16.17, *p* < 0.01; equation Y = 0.4717X + 0.6105, R^2^ = 0.4247). Immunofluorescence revealed that SD stress produced a significant increase in the number of Fos-positive cells in the BLA (Fig. 3e and f, Student’s *t*-test, *t*_*13*_ = 2.635, *p* < 0.05).

**Fig. 4:**
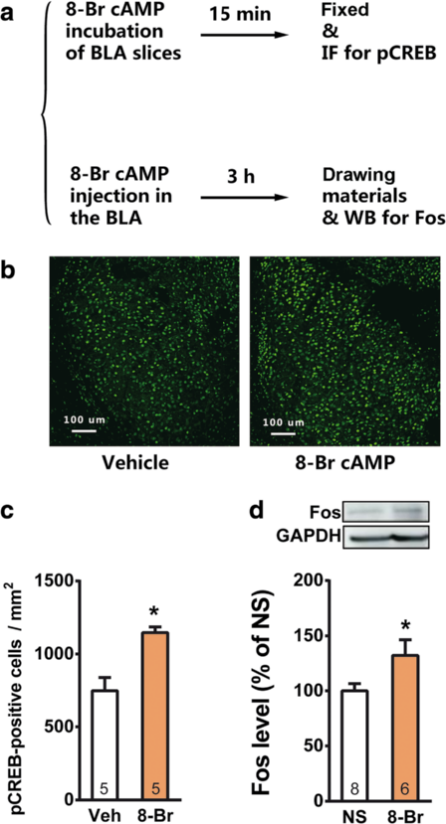
PKA activation induced pCREB and Fos expression in the BLA of naïve mice. **a** Schematic representation of *in vitro* and *in vivo* protocols to determine pCREB and Fos expression; **b** & **c** Preincubation of amygdala slices with 8-Br-cAMP (8-Br) increased the pCREB-positive cells in the BLA; **d** Injection of 8-Br-cAMP in the BLA upregulated Fos level in the BLA. *p < 0.05 verse vehicle control.

To further determine the role of PKA in the CREB activation and Fos expression, we delivered two protocols probing into the two molecules in *vitro* and in *vivo*, respectively (Fig. 4a). Preincubation of amygdala slices with 8-Br-cAMP (5 mM) [32], a membrane-permeable cAMP analogues, for 15 min induced substantial elevation of p-CREB in the BLA (Fig. 4b and c, Paired *t-*test *t*_8_ = 4.501, *p* < 0.05). PKA activation also induced Fos expression *in vivo*: intra-BLA injection of 8-Br-cAMP (2 μg) [33, 34] significantly increased Fos expression level in the BLA (Fig. 4d, Student’s *t*-test, *t*_*12*_ = 2.3393, *p* < 0.05).

### Activation of PKA in the BLA antagonizes social defeat-induced anxiety but not social avoidance

**Fig. 5:**
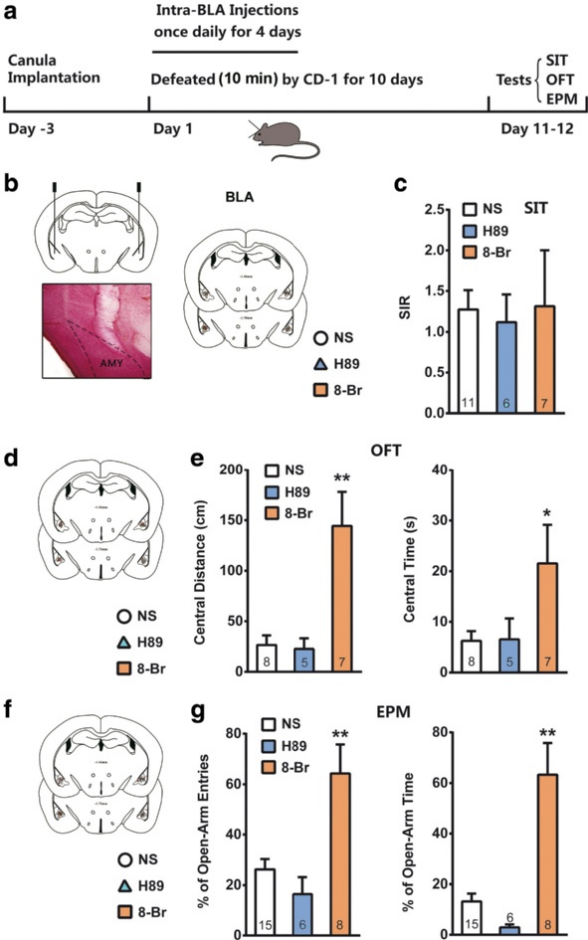
Activation of PKA in the BLA antagonized social defeat-induced anxiety but not social avoidance. **a** Schematic representation of a protocol for 4-day H-89/8-Br-cAMP administration during the SD stress; **b** & **c** The PKA activator 8-Br-cAMP (8-Br) and inhibitor H-89 did not affect the social interaction behaviors; **d**-**g** 8-Br-cAMP alleviated SD-induced anxiety in the OFT (**d** & **e**) and EPM test (**f** & **g**). **b**, **d** and **f** are reconstruction of microinjection sites of the bilateral BLA in different behavioral tests. Histological photomicrograph shows cannula placement in the BLA. *p < 0.05, **p < 0.01 verse NS control.

**Fig. 6:**
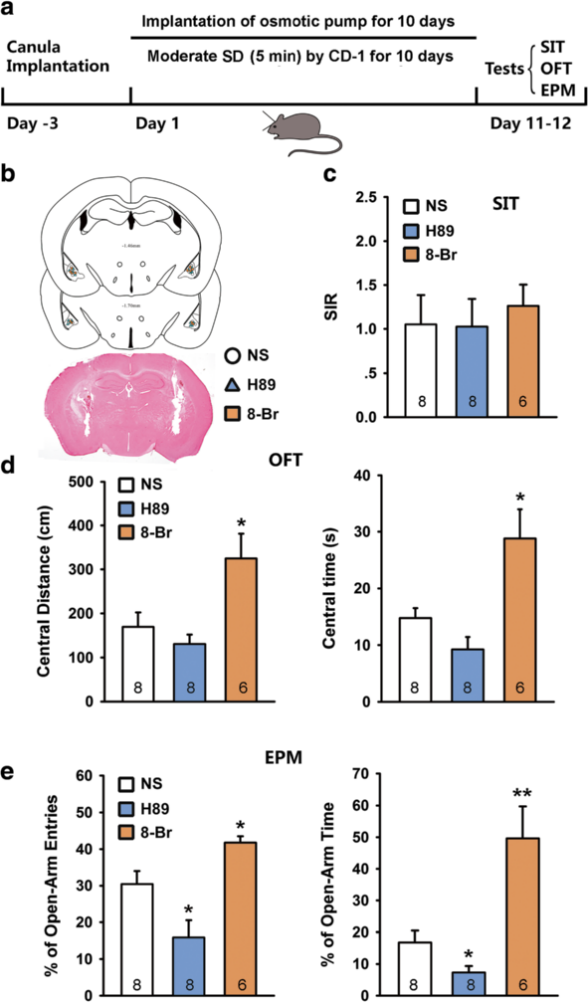
Inhibition of PKA in the BLA aggravated moderate social defeat-induced anxiety but not social avoidance. **a** Schematic representation of a protocol for 10-day H-89/8-Br-cAMP administration during a moderate SD stress (the exposure time to aggressors was shortened from 10 min to 5 min each day); **b** photomicrograph of coronal section and histological reconstruction of microinjection sites by osmotic pump system in the bilateral BLA; **c** Continuous PKA activator 8-Br-cAMP (8-Br, 2 μg/d) and inhibitor H-89 (0.3 μg/d) over the 10-day defeat did not affect the social interaction behaviors; **d** & **e** 8-Br-cAMP alleviated moderate SD-induced anxiety in the OFT (**d**) and EPM test (**e**), whereas H-89 significantly aggravated moderate SD-induced anxiety in the EPM test. *p < 0.05, **p < 0.01 verse NS control.

**Fig. 7:**
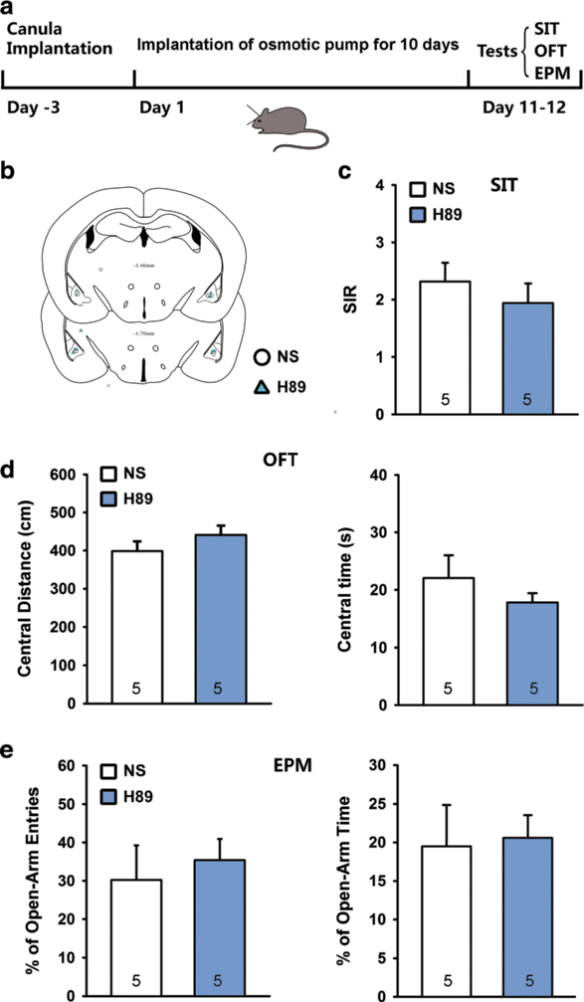
Inhibition of PKA in the BLA did not change social interaction, open field and elevated plus maze behaviors in naïve mice. **a** Schematic representation of the protocol for 10-day H-89 (0.3 μg/d) continuous administration; **b** Reconstruction of microinjection sites in the bilateral BLA in following behavioral tests; **c**-**e** Continuous PKA inhibitor H-89 (0.3 μg/d) by osmotic pump system for 10 days did not affect the social interaction (**c**) OFT (**d**) and EPM (**e**) behaviors.

To determine the contribution of PKA in the SD-induced anxiety and social avoidance, we made repeatedly bilateral intra-BLA administrations of PKA agonist 8-Br-cAMP (2 μg) [33, 34] or PKA antagonist H89 (0.3 μg) [35], 1 h before attacks during the first 4 days of a 10-day SD stress (Fig. 5a). Neither H89 nor 8-Br-cAMP affected the social behavior of SD exposed mice in SIT (Fig. 5b and c, one-way ANOVA, F_2, 21_ = 0.4985, *p* > 0.05). Interestingly, 8-Br-cAMP significantly alleviated the SD stress-induced anxiety-like behaviors in OFT and EPM test (Fig. 5d-g, one-way ANOVA, *p* < 0.05 or *p* < 0.01). However, inhibition of PKA by H89 did not substantially affect SD stress-induced anxiety-like behaviors. Considering that the effect of 4-day 8-Br-cAMP/H-89 infusion on defeat behaviors could disappear or result in compensation after cessation of intra-BLA injections for 6 days, we examined the effects of 10-day 8-Br-cAMP/H-89 infusion during the 10-day defeats. To prevent floor effect, we used a moderate SD stress protocol by shortening exposure time to aggressors from 10 min to 5 min (Fig. 6a, Additional file 1: Figure S1). 8-Br-cAMP and H-89 was delivered in a dosage of 2 μg/d and 0.3 μg/d respectively by a micro-osmotic pump system during the 10-day SD period. The behaviors were tested 24 h after removing the pumps. Consistently, both H89 and 8-Br-cAMP did not change social behavior of SD exposed mice in SIT (Fig. 6b and c, one-way ANOVA, F_2, 19_ = 0.873, *p* > 0.05). Also, 8-Br-cAMP significantly alleviated the moderate SD stress-induced anxiety-like behaviors in OFT and EPM test (Fig. 6d and e). Interestingly, 10-day H-89 infusion into the BLA significantly worsened moderate SD stress-induced anxiety-like behavior in EPM test (Fig. 6e). Although the central distance and central time were decreased in OFT following H-89 treatment, no significant difference was observed (Fig. 6d). Intra-BLA infusion of 10-day H-89 had no effect on social interaction, open field and elevated plus maze behavioral tests in naïve mice (Fig. 7).

**Fig. 8:**
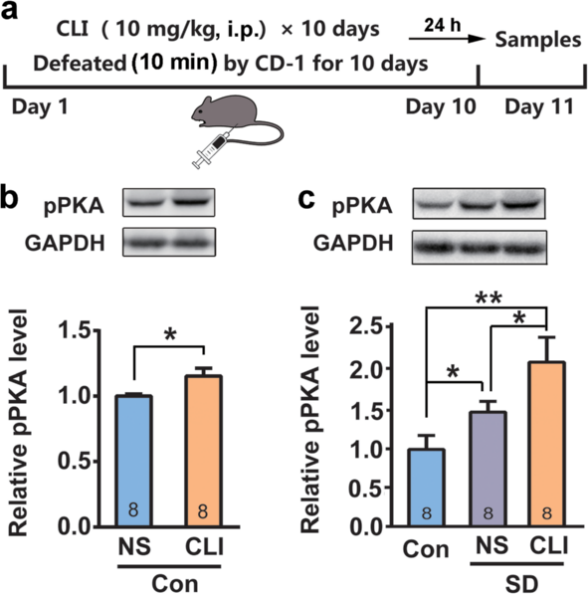
Clomipramine (CLI) activated amygdaloid PKA. **a** Schematic representation of the protocols of CLI administration during SD stress and sampling; **b** & **c** CLI increased pPKA level in the BLA of the control (**b**) and SD stress mice (**c**). *p < 0.05, **p < 0.01.

These results suggest that PKA activation in the BLA exerts an antixiolytic effect in SD exposed animals. We speculate that the increased pPKA level in the BLA might be the result of functional compensation during the SD stress-induced anxiety development. To confirm the above assumption, we examined the effect of antidepressant CLI on amygdaloid pPKA. As shown in Fig. 8, repeated injections of CLI (10 mg/kg, i.p.) in control mice markedly increased pPKA level in the BLA (Fig. 8b, Student’s *t*-test, *t*_14_ = 2.519, *p* < 0.05). In particular, repeated CLI treatment in SD exposed mice produced a much higher pPKA level in the BLA (Fig. 8c, one-way ANOVA, F_2, 21_ = 10.692, *p* < 0.01). This data not only strongly support the view that PKA activation is a compensatory instead of pathogenic response but also have implications in the mechanisms underlying the anti-anxiety of CLI.

## Discussion

The present study demonstrates that exposure to 10 days of SD induces significant social avoidance and anxiety-like behaviors, consisting with the previous studies using this stress paradigm. Meanwhile, PKA and its downstream targets CREB and Fos were activated in the amygdala. Blockade of PKA activation in the BLA by H-89 aggravated moderate SD-induced anxiety-like behavior in EPM test, and the BLA infusion of PKA agonist 8-Br-cAMP significantly antagonized SD-induced anxiety-like behaviors, suggesting that PKA activation in the amygdala actually plays a counter-stress rather than pathogenic role. Furthermore, antidepressant CLI treatment significantly ameliorated anxiety-like behavior and elevated pPKA level in the BLA. Thus, the pPKA upregulation in the BLA by SD stress in the present study might be a functional compensation during the development of SD stress-induced anxiety.

Although amygdaloid PKA activation has been linked to anxiety and depression, results are not always consistent depending on the differences in animal species/strains, models, test periods and behavioral test paradigms. For examples, Chen et al. reported that chronic social defeat for 4 weeks in Fischer 344 rats significantly reduced protein levels of PKA in the amygdala [36]. With higher basal and stimulated PKA activity levels in the basolateral amygdala, *Prkar1*α^+/−^ mice exhibited anxiety- and depression-like behaviors, suggesting that an increase in PKA activity may be associated with an increased risk for anxiety [24]. However, opposite outcomes showed that deficiency of RCS (regulator of calmodulin signaling), a PKA-regulated phosphoprotein that is high expression in the dorsal striatum and amygdala, displayed an enhanced anxiety response [37]. As a support, our current study demonstrated that PKA activator in the BLA effectively alleviated the SD stress-induced anxiety-like behaviors in OFT and EPM test, indicating an anxiolytic effect of PKA activation. Furthermore, PKA inhibitor in the BLA produced an aggravated anxiety-like behavior in EPM test, although there were no differences in the OF test. Give that the EPM test explores the conflict between the natural behavior of rodents to explore new spaces and avoid open spaces, it is considered to be a more sensitive test of anxiety [38–40].

The transcription factor CREB is one of the most studied molecules relevant to anxiety and represents an important linker between a number of neurotransmitters and downstream gene expression [22, 30]. CREB is regulated via phosphorylation by PKA, CaMK-IV and MAPKs [41]. It is well known that *c-fos* is one of the first identified CREB target genes, whose expression is induced in a CRE-dependent manner in response to certain stimuli that activate CREB [31]. Fos family are used as markers of neuronal activity and can be modulated by stress [42, 43]. A study from Bourne et al. showed that socially defeated mice displayed elevated levels of Fos positive cells in the BLA [16]. FosB upregulation in the BLA was also observed in nerve injury mice who exhibited anxiety-like behaviors [44]. Consistently, we further demonstrated a correlation between increased pPKA and expression of pCREB and Fos in the BLA following SD stress.

It is worth mentioning that activation of PKA in the BLA did not affect social behaviors of SD-treated mice, suggesting a relatively independent relationship of the brain regions responsible for anxiety and social avoidance. Several studies showed that the nucleus accumbens (NAc) excitability might be associated to the SD stress-dependent social behaviors [45–47]. Our preliminary data showed that CREB activation in the nucleus accumbens (NAc), especially the shell of NAc might be involved in resiliency to social defeat stress. Resilient defeated mice showed a higher pCREB expression in the shell of NAc than that of susceptible ones (Additional file 2: Figure S2).

## Conclusions

In summary, SD stress induces two major types of behavioral defects, social avoidance and anxiety; PKA activation in the BLA responses this stress as an anti-anxiety role; social avoidance could not be directly modulated by amygdaloid PKA.

## Methods

### Animals

Adult male C57BL/6J mice (8 weeks old) were bought from Shanghai Experimental Animal Center of the Chinese Academy of Sciences. Animals were housed in a temperature- and humidity-controlled room with a 12:12 light-dark cycle (lights on 07:00) and free access to food and water. CD-1 mice retired breeders (male, 8-10 months) from Vital River Laboratories (Beijing, China) were used as the aggressors. The aggressors were screened every 3 months to ensure their antagonistic interactions. To control for possible effects of time of day, mice were trained and tested at approximately the same time of day (light period). All experimental protocols and animal handling procedures were permitted by the Experimental Animal Ethics Committee of Shanghai Medical College, Fudan University. All the following behavioral tests were performed by the same experimenter blinded to the group assignment to minimize the differences between-experimenters. Some behavioral tests were performed in the same animals during two consecutive days. Day 1 started with social interaction test followed by open-field test with a rest for 4-6 h in home cages. On day 2, elevated plus maze test was performed.

### Social defeat stress

Repeated social defeat (SD) stress was applied as described previously [7]. Briefly, the C57BL/6J mouse was individually introduced into the home cage of an unfamiliar resident aggressor CD-1 mouse for 5-10 min of direct contact, during which it was attacked and displayed subordinate posturing, and then housed together with the CD-1 (separated by a perforated plastic divider which allowed olfactory, visual, and auditory communication but prevented tactile contact) for the remaining 24 h. During a whole period of SD stress, the experimental mouse was subjected to social defeat for 10 consecutive days using 10 different CD-1 aggressors (Additional file 1: Figure S1).

### Social interaction test

Social interaction behavior was assessed by social interaction test (SIT) on day 11 (24 h after the 10^th^ defeat by the CD-1 mouse) in an open-field apparatus (42 cm × 42 cm). Each test consisted of two 2.5-min sessions separated by an interval of 30–60 s. In the first session, the experimental mouse was introduced into the open-field with a small empty mesh cage (10 cm × 6 cm) on one wall (target absent), and in the second session, a similar cage containing an unfamiliar CD-1 mouse (target present). The time in the interaction zone (IZ) that covered 8 cm around the mesh cage (14 cm × 26 cm) was defined as the interaction duration. A social interaction ratio (SIR) was calculated by interaction duration on CD-1 (2^nd^ session)/interaction duration on empty cage (1^st^ session).

### Open field test

The open field test (OFT) apparatus consists of an open box (50 cm × 50 cm × 40 cm, length × width × height), which was evenly illuminated to 15 lux. Mice were individually placed into the center of the arena and allowed to explore for 5 min. Activity was videotracked and the time spent and distance travelled in the central zone (covering 25 cm × 25 cm) were recorded.

### Elevated plus maze test

The elevated plus maze (EPM) test consists of four arms (5 × 30 cm). Two closed arms have 20-cm-high walls and the other arms are left open (open arm). The maze was elevated 40 cm above the floor. Light intensities in the central area, opened and closed arms were set to 15, 15 and 5 lux, respectively. Mice were placed in the center of the maze facing an open arm, and allowed free access to 4 arms for 5 min. The number of entries into open arms and time spent in open arms were recorded. Percentage of open-arm entries ([open entries]/[total entries] × 100) and open-arm time ([time in open arms]/[time in total arms] × 100) were calculated.

### Rotarod test

One day before the test, the mice were habituated and trained 4 times to run the rotarod (IITC Life Science) for 1 min at a fixed speed of 12, 16, 20, 24 rpm, respectively. The intertrial interval was 2 min. On the day of experiment, coordination was evaluated by increasing rotation cycle from 12 to 24 rpm for 1 min. Rotarod data were expressed as the average latency to fall of three sessions for each animal.

### Intra-amygdala drug infusions

Mice were anesthetized with intraperitoneal sodium pentobarbital (45 mg/kg), and then securely placed into a stereotaxic device with bregma and lambda at a horizontal level. A 30 gauge stainless steel guide cannula with a 33 gauge stainless steel stylet plug (Reward, Shenzhen, China) was bilaterally implanted 0.5 mm above the BLA injection site [anteroposterior (AP) −1.6 from bregma, mediolateral (ML) ± 2.9, dorsoventral (DV) −4.5 from the cranium surface] according to the atlas of Paxinos and Franklin (1997). The cannula was fixed to the cranium with denture acrylic cement. Animals were allowed to recover for 3-4 days before next experimental procedure. At the end of the experiment, brains were sectioned for neutral red staining to verify the cannula position and injection sites.

Microinjection was performed through a 33 gauge stainless steel injection cannula that extended 0.5 mm beyond the tip of the guide cannula. The injection cannula was connected to a 1 μl Hamilton syringe. A total volume of 0.5 μl per BLA of either vehicle or drug was injected over a 5 min period. The injection cannula was left in place for an additional 2 min to minimize spread of the drug along the injection track.

To examine the effect of PKA on the social avoidance and anxiety-like behaviors, repeated intra-BLA microinjections of vehicle (normal saline), H-89 (0.3 μg per side, a PKA inhibitor) [35] or 8-Br-cAMP (2 μg per side, a PKA activator) [33, 34] were performed 1 h before attacks during the first 4 days of a 10-day repeated SD stress. The behaviors were tested 24 h after the 10-day SD stress. To examine the effect of PKA activation on BLA Fos expression, 8-Br-cAMP (2 μg per side) or vehicle was microinjected into the bilateral BLA of the naïve mice. After 3 h, mice were sacrificed and the BLA samples were collected.

The other method was continuous BLA delivery using micro-osmotic pumps. Implantation of the bilateral BLA cannula was performed as described above. An micro-osmotic pump (model 1002; ALZET, Cupertino, CA, USA) with a delivery rate of 0.25 μl/h during 10 days, connected via polyethylene tubing to a brain infusion kit (model ALZET Brain Infusion Kit II, ALZET), was used to infuse H-89 (5 μg/100 μl), 8-Br-cAMP (33 μg/100 μl) or saline to the mouse bilateral BLA. H-89 and 8-Br-cAMP was delivered in a dosage of 0.3 μg/d and 2 μg/d during the 10-day SD period days, respectively. The behaviors were tested 24 h after removing the pumps.

### Western blotting

After defined survival times, mice were sacrificed by overdose anesthetic and the brain was removed quickly. The basolateral amygdala (BLA) was dissected on ice using a mouse Brain Matrix (Stoelting Company). Briefly, one coronal brain slice (1 mm thick) containing BLA (AP −0.8 ~ −1.8) was cut and the bilateral amygdaloid tissues were dissected using a surgical blade according to the altas of Paxinos and Franklin (1997). The samples were rapidly frozen in liquid nitrogen. Frozen samples were homogenized in a lysis buffer containing a mixture of protease inhibitors (Roche) and PMSF (Sigma), and were centrifuged at 10,000 rpm for 15 min at 4 °C. The supernatants were used for western blotting.

Equal amount of protein (~20 μg) was load and separated in 8 % Tris-Tricine SDS-PAGE gel (Bio-Rad). The resolved proteins were transferred onto PVDF membranes (Millipore). The membranes were blocked in TBST-milk (TBS, 0.1 % Tween 20, 5 % skim milk) for 1 h at 37 °C, and then incubated overnight at 4 °C with primary antibodies [rabbit anti-phospho-PKA (pPKA) Substrate (1:1000, Cell Signaling) or mouse anti-pPKA RII (1:1000, Upstate Biotechnology); rabbit anti-pCREB (1:1000, Millipore) and rabbit anti-Fos (1:1000, Santa Cruz Biotchnology)]. The blots were then incubated with the secondary antibody, goat anti rabbit or goat anti mouse IgG conjugated with HRP (1:10000, Pierce) for 2 h at room temperature (RT). GAPDH antibody was probed as a loading control. Signals were visualized using enhanced ehemiluminescence (Pierce), and captured by ChemiDoc XRS system (Bio-Rad). A Bio-Rad image analysis system was then used to measure the integrated optic density of the specific bands.

### Immunohistochemistry

Mice were deeply anesthetized and were transcardially perfused with normal saline followed by 4 % paraformaldehyde in 0.1 M PB (pH 7.4). Brains were removed, postfixed in the same fixative overnight, and immersed in 20 % and 30 % sucrose (0.1 M PBS) for 24 ~ 48 h at 4 °C for cryoprotection. Coronal sections were cut at 30 μm with a cryostat (CM1850, Leica Microsystems). For immunofluorescence, free-floating sections were blocked by 5 % donkey serum in 0.01 M PBS with 0.3 % Triton-X100 for 1 h at RT. Sections were then incubated overnight at 4 °C with primary antibodies [rabbit anti- Fos (1:200, Cell Signaling) and rabbit anti-pCREB (1:1000)]. The sections were then incubated for 2 h at RT with Alexa Fluor 488-conjugated secondary antibody (donkey anti-rabbit, 1:200, Life Technologies). The sections were coverslipped, and then observed with a confocal laser scanning microscope (model FV1000, Olympus). For the quantification of immunoreactive signals, four non-adjacent sections from each mouse through the BLA were randomly selected. The numbers of pCREB- and Fos-labeled cells were counted in the BLA that was captured inside the optic field.

### Brain slice preparation

Coronal brain slices containing the BLA were obtained from naïve mice. After anesthetizing, mice were decapitated. The brain was rapidly removed and immersed in preoxygenated (95 % O_2_, 5 % CO_2_) cold artificial CSF (ACSF) containing (mM): 234 sucrose, 2.5 KCl, 1.25 Na_2_H_2_PO_4_, 28 NaHCO_3,_ 7 MgCl_2_, 0.5 CaCl_2_, 7 glucose, 1 ascorbic acid. The osmolarity was adjusted to 300 mosmol/L and the pH 7.4. Slices (250 μm) were cut with a vibratome (VT 1000S, Leica, German) and transferred to an oxygenated chamber at RT for at least 45 min before further processing. For immunohistochemical experiments, the amygdaloid slices were treated with 8-Br-cAMP (5 mM) for 15 min at RT. Adjacent untreated slices served as controls. The slices were rapidly fixed by cold 4 % paraformaldehyde for 3 h, and then processed for immunofluorescence staining.

### Statistical analysis

Data are presented as mean ± SEM. Student’s *t*-test and one-way ANOVA followed by Newmann-Keuls *post hoc* test were used to identify significant differences. Linear regression was applied to analysize the relationship between pPKA and pCREB. In all cases, *p* < 0.05 was considered to be statistically significant.

## Additional files

Additional file 1: Figure S1. Social defeat paradigm. (TIF 442 kb) Additional file 2: Figure S2. SD Stress activated CREB in the nucleus accumbens (NAc). (TIF 848 kb)
