# Supplementary figures and images for: Activation of protein kinase A in the amygdala modulates anxiety-like behaviors in social defeat exposed mice
Source: Mol Brain. 2016 Jan 8;9:3. doi: 10.1186/s13041-015-0181-3 (PMC4706664; doi:10.1186/s13041-015-0181-3)

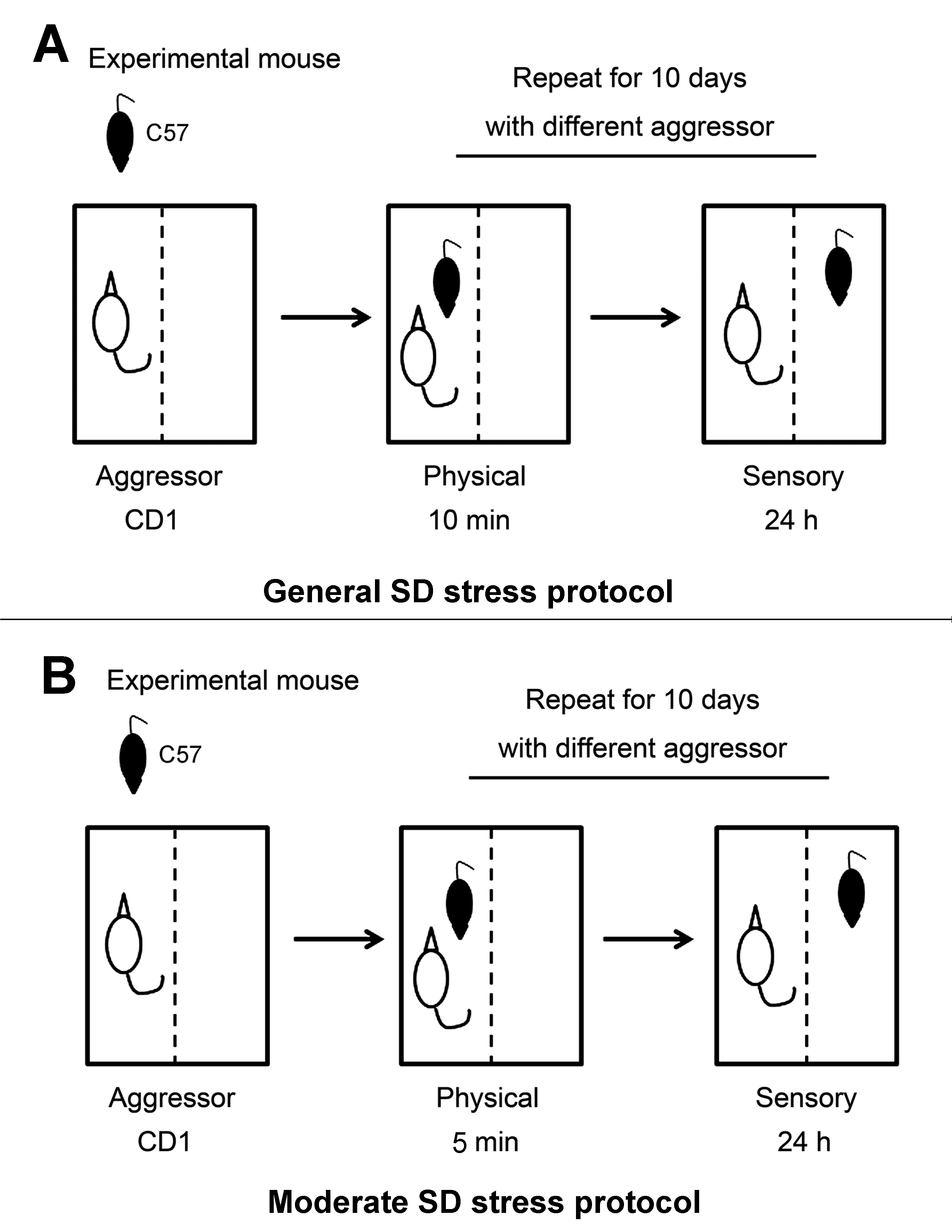

Supplement: Additional file 1: Figure S1. — Social defeat paradigm. (TIF 442 kb) [file 13041_2015_181_MOESM1_ESM.tif]

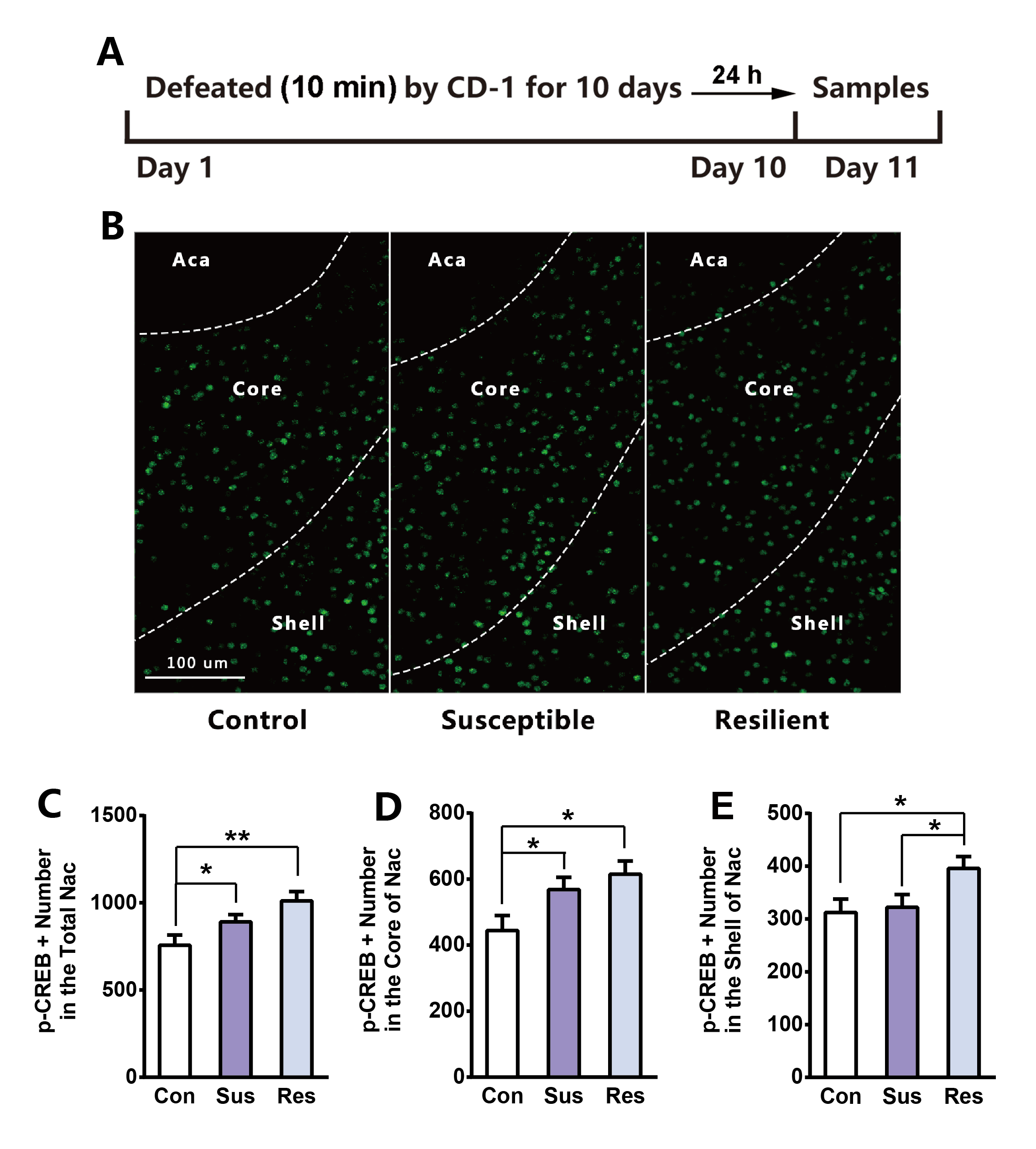

Supplement: Additional file 2: Figure S2. — SD Stress activated CREB in the nucleus accumbens (NAc). (TIF 848 kb) [file 13041_2015_181_MOESM2_ESM.tif]
